# Bioactive compound C498-0670 alleviates LPS-induced sepsis *via* JAK/STAT and NFκB signaling pathways

**DOI:** 10.3389/fimmu.2023.1132265

**Published:** 2023-04-14

**Authors:** Jing Xu, Xinxin Zhang, Mingming Zhou, Peizhe Lu, Yuting Xu, Lihong Wu, Qianyue Zhang, Zhihua Wu, Xiaoyu Xu, Pengfei Shi, Qingda Wei, Xiaoyu Li, Qiaoling Song

**Affiliations:** ^1^ Department of Gastroenterology, The Affiliated Hospital of Qingdao University, Qingdao, China; ^2^ Innovation Platform of Marine Drug Screening & Evaluation, Qingdao Pilot National Laboratory for Marine Science and Technology, Qingdao, Shandong, China; ^3^ Department of Neuroscience, University of Michigan, Ann Arbor, MI, United States; ^4^ Key Laboratory of Marine Drugs, Ministry of Education of China, School of Medicine and Pharmacy, Ocean University of China, Qingdao, China; ^5^ College of Marine Life Sciences, Ocean University of China, Qingdao, China; ^6^ School of Medicine, Zhengzhou University, Zhengzhou, China

**Keywords:** anti-inflammatory, JAK/STAT, NFκB, LPS, transcriptome sequencing, septic shock

## Abstract

The JAK/STAT and NFκB signaling pathways are two major inflammatory signaling pathways that are usually activated simultaneously in the body’s inflammatory response to bacterial or viral infections. Hyperactivation of these two prominent signaling pathways is associated with various immune-related diseases and mortality, pointing to an urgent need for drug development targeting JAK/STAT and/or NFκB signaling. In this study, we screened 18,840 compounds using our well-established dual STAT-NFκB driven luciferase reporter based high-throughput screening system and identified a bioactive compound C498-0670, which inhibits both JAK/STAT and NFκB signaling. C498-0670 inhibits the activation of STATs and p-IKKα/β in both the immortalized cell lines and primary peritoneal macrophages, while suppressing the expression of LPS-induced inflammatory mediators *in vitro*. In addition, the overall anti-inflammatory effects of C498-0670 were investigated using transcriptome sequencing and bioinformatics approaches. C498-0670 was predicted to alleviate sepsis/septic shock by disease/function analysis using IPA software, which was further verified in the LPS-induced mouse sepsis model *in vivo*. C498 reduced LPS-induced liver and kidney damage, myeloid cell infiltration, and pro-inflammatory cytokine and chemokine production *in vivo*. Furthermore, the SPR-HPLC-MS-based target fishing approach was used to identify the putative drug targets, and the high affinities of JAK2 (JAK/STAT signaling), NFKBIA (NFκB signaling), and IL-1β, NLRP1b (inflammasome signaling) for C498-0670 were verified by molecular docking approach. These results suggest that C498-0670 can be used as a dual-target inhibitor of JAK/STAT and NFκB signaling pathways for the treatment of various inflammatory diseases, especially septic shock.

## 1 Introduction

Inflammation, especially acute inflammation, is the human body’s defense response to various infections, harmful stimuli, and tissue damage (1). However, an over-activated immune system can lead to organ damage or even death. The secretion of a large number of cytokines such as interleukin 1 (IL-1), interleukin 6 (IL-6), tumor necrosis factor alpha (TNF-α), and the overactivation of the related signaling pathways including JAK/STAT and NFκB could cause serious side effects such as vasodilation, increased permeability, leukocyte exudation and ultimately tissue destruction (2). For example, excessive IL-6 production could induce cell apoptosis and lung injury by activating the JAK2/STAT3 signaling pathway (3). Activation of the NFκB pathway increases the transcription of inflammation-related genes and induces increased secretion of cytokines such as IL-6 and TNF-α, leading to pathological damage in patients (4). Therefore, the development of drugs targeting the JAK/STAT and NFκB pathways may be a promising strategy for the treatment of inflammatory diseases.

JAK/STAT signaling begins with the binding of various ligands such as growth factors, interleukins or interferons to the specific membrane receptors to activate JAK/STAT cascades (5). For instance, IL-6 binds to the IL-6R/gp130 complex to recruit JAKs (JAK1/2 and TYK2) intracellularly, which further phosphorylates STAT3, allowing it to enter the nucleus, which induces the production of its downstream genes such as IL-1β and IL-6. Type I interferon (IFN-I) binds to the interferon-α/β receptor (IFNAR), phosphorylates downstream STAT1 and STAT2 *via* activated TYK2 and JAK1, and binds to the IFN regulatory factor 9 (IRF9) to form the IFN-stimulated gene factor 3 (ISGF3) complex. The ISGF3 complex then translocates to the nucleus and participates in the inflammatory response (6). Similarly, NFκB signaling can be activated by a variety of ligands, such as Toll-like receptor (TLRs) agonists and cytokines (TNF-α and IL-6), and downstream mediators, including IKK and IκB during the inflammatory response. TNF-α binds to the TNF receptor and interacts with intracellular mediators to transmit the signal to IκB kinase (IKK). IκB is then dissociated from the p50/p65/IκB heterotrimer and degraded by the proteasome to release NFκB, which enters the nucleus to regulate the release of pro-inflammatory factors (7). Gram-negative bacterial components such as LPS bind to TLR4 and activate the myeloid differentiation factor 88 (MyD88) dependent IKK-IκB-NFκB cascade to trigger the production of pro-inflammatory cytokines such as TNF-α and IL-1 (8). Meanwhile, LPS activates TLR4 mediated TIR-domain-containing adapter-inducing IFN-β (TRIF)-dependent IRFs’ activities to induce IFN-I production, which could activate JAK/STAT signaling to drive the expression of downstream ISGs (9). In addition, the activation of both TRIF and Myd88 signaling could induce IL-6 expression, which could subsequently activate STAT3 signaling. Therefore, mechanistically, JAK/STAT and NFκB signaling pathways could co-activate during the infection process such as in bacterial infection, and co-operatively and complementarily exert inflammatory stimulating effects. Besides, once either signaling pathway is activated by stimuli such as endotoxins and cytokines, cytokines and chemokines can be induced to activate another signaling pathway and cause the sequential activation of JAK/STAT and NFκB signaling pathways (10, 11). Furthermore, NFκB family members RelA/p65 and p50 could directly and physically interact with STAT3, leading to specific transcriptional synergy, which explains the intense interaction of JAK/STAT and NFκB signaling pathways (11). Therefore, although inhibitors of both JAK/STAT and NFκB signaling pathways are under continuous investigation (12, 13), inhibitors that target both JAK/STAT and NFκB signaling pathways may be more promising for anti-inflammatory treatment (14).

Sepsis is widely defined as a life-threatening organ dysfunction (15) and is a syndrome of multiple organs or tissue damage caused by a systemic inflammatory response. The JAK-STAT signaling pathway plays a key role in sepsis and is involved in both the systemic inflammatory response syndrome (SIRS) and the compensatory anti-inflammatory response syndrome (CARS) phases of sepsis (16). Similarly, MicroRNAs promote sepsis-induced cardiomyopathy progression and neurovascular dysfunction by upregulating genes associated with the NFκB signaling pathway (17). In the experimental models of sepsis, JAK2 inhibitors have been shown to block the classical p65RelA/p50 NFκB pathway, inhibiting the production of inflammatory factors, thus saving animals from sepsis (18). Either alone or in concert, the JAK/STAT and NFκB pathways play a critical role in the progression of severe inflammatory diseases such as sepsis. Therefore, dual-target inhibitors would be more effective for the treatment of inflammatory diseases.

In the current study, to identify the drug hits for these two pathways, we performed a high-throughput drug screening process of 18,440 small molecule compounds using our established luciferase based dual-JAK/STAT and NFκB signaling drug screening platform (14). A novel bioactive compound C498-0670 (C498), which was designed as a microtubule-associated protein TAU inhibitor, was identified as a dual-JAK/STAT and NFκB inhibitor. Both *in vitro* experiments and transcriptome analysis indicate that C498 effectively inhibited the activation of STATs and IKK, and the LPS induced gene expression changes. Functional analysis also suggested that C498 had the potential to treat various inflammatory diseases including sepsis. In an *in vivo* LPS induced sepsis model, C498 ameliorated the reduction of body temperature and inhibited the systemic inflammatory response and tissue damage caused by LPS. Besides, through the target fishing approach and molecular docking analysis, targets putatively responsible for the inhibition of JAK/STAT (JAK2), NFκB (NFKBIA) and inflammasome formation (IL-1β and NLRP1b) were identified and validated. All these data suggest that C498 may serve as a promising anti-inflammatory drug for the treatment of various inflammatory diseases.

## 2 Materials and methods

### 2.1 Antibodies and reagents

Primary antibodies used in the study were detailed in **Table S1**. Cell lysis buffer (Cat. 9803) was purchased from Cell Signaling Technology. Protease Inhibitor (Cat. 11836145001), Phosphorylation Inhibitor Cocktail Reagent (Cat. 4906837001) and SYBR Green PCR Master Mix (2×) (Cat. 4913914001) were from Roche Diagnostics. Horseradish peroxidase-conjugated secondary antibodies (Cat. abs20001; abs20002) were obtained from Absin. Kolliphor^®^ HS 15 (HS-15) (Cat. 42966) and lipopolysaccharide/LPS (Cat. 916374) were from Sigma. Recombinant mouse IL-6 (Cat. 200-06), human and mouse TNF-α (Cat. 315-01A; 300-01A), mouse IFN-γ (Cat. 315-05) and IFN-β (Cat. 300-02BC) were obtained from PeproTech. Mouse granulocyte colony-stimulating factor (G-CSF) ELISA Kit (Cat. ab197743) was bought from Abcam. Mouse IL-6 ELISA Kit (Cat. abs520004) and Mouse IL-1β ELISA Kit (Cat. abs520001) were purchased from Absin. Urea Assay Kit (Cat. C013-2-1), Alanine aminotransferase (ALT) Assay Kit (Cat. C009-2-1), and Aspartate aminotransferase (AST) Assay Kit (Cat. C010-2-1) were purchased from Nanjing Jiancheng Bioengineering Institute. The detailed compound library information was as in our previously published literature (14). The bioactive compound C498-0670 (C498) was acquired from TargetMol.

### 2.2 Cell culture

DU145, A549, THP-1, and HeLa cells were purchased from the American Type Culture Collection (ATCC). The established dual STAT-NFκB driven luciferase reporter cell line SKA-II was derived from A549 cells, which were transfected with luciferase expressing plasmid containing a 16 × SIE (8 × 5’-TTCCTGTAA-3’ and 8 × 5’-TTCCCGTAA-3’) plus 1 × NFκB (5’-GGGAATTTCC-3’) binding element with one TATA box (14). HeLa, SKA-II cells (14), and peritoneal macrophages were incubated in Dulbecco’s modified Eagle’s medium (DMEM), and DU145 and THP-1 cells were cultured in Roswell Park Memorial Institute (RPMI) 1640 medium. Cells were incubated in a culture medium supplemented with 10% fetal bovine serum (FBS, Gibco), 100 IU/ml penicillin, and 100 μg/ml streptomycin. All cells were cultured at 37°C in humidified incubators with 5% CO_2_. All the cell lines were authenticated by STR profiling and tested without mycoplasma contamination.

### 2.3 Animals

Male C57BL/6 strain mice (SPF degree, 6-8 weeks old) were purchased from Beijing Vital River Laboratory Animal Technology Co., Ltd. They were housed in a laboratory animal room free of specific pathogens with a 12 hours light-dark cycle. All animal experiments were approved by the Committee of Experimental Animals of the School of Medicine and Pharmacy, Ocean University of China (OUCSMP-20220301).

### 2.4 Primary peritoneal macrophage isolation

Detailed protocol for harvesting primary mouse peritoneal macrophage has been reported previously (19). Briefly, 38.5 grams of the BBL™ Thioglycollate Medium Brewer Modified powder (BD Biosciences, Cat. 211716) was dissolved in 1 L purified water under frequent agitation, autoclaved at 121°C for 15 min and then stored at 4°C for at least 3 months. Mice were injected intraperitoneally with 1 ml of aged thioglycolate. Mice were sacrificed 72 hours later, and peritoneal macrophages were harvested by flushing the peritoneal cavity.

### 2.5 Luciferase reporter assay

SKA-II cells (8,000/well) were inoculated into 96-well white plates (Corning) and incubated overnight at 37°C in an incubator with 5% CO_2_. These cells were then treated with either vehicle or C498 at the indicated concentrations for 24 h. Luciferase activity was determined using Promega luciferase kits (Cat. E2510) and measured by a SpectraMax^®^ L microplate reader (Molecular Devices). The *in vitro* luciferase experiments were repeated three times.

### 2.6 Western blotting

The immortalized cell lines and peritoneal macrophages were treated with 2.5, 5, 10, and 15 μM of C498 for 2 h followed by cytokine stimulation: 20 ng/ml IL-6, 50 ng/ml IFN-β/IFN-γ, or 20 ng/ml TNF-α for 10 min, or 100 ng/ml LPS for 0.5 h. Cells were washed twice with cold PBS and harvested in cell lysis buffer containing protease and phosphatase inhibitors. A total of 20 μg protein lysates were resolved on SDS-PAGE electrophoresis gel and transferred onto nitrocellulose (NC) membranes (GE Healthcare, Cat. 10600034). After blocking with 5% nonfat milk solution in TBST (10 mM Tris, pH 8.0, 150 mM NaCl, 0.1% Tween 20) for 1.5 h, the membranes were incubated with primary antibodies overnight at 4°C. After being washed with TBST, the membranes were incubated with horseradish peroxidase-conjugated secondary antibodies for 2 h at room temperature. Immune complexes were detected with an Immobilon™ Western Chemiluminescence HRP Substrate (Millipore, Cat. NO. WBKLS0500) and photographed with a Tanon 5200 imaging system. The *in vitro* Western blotting experiments were repeated three times.

### 2.7 Real-time PCR measurement

Total RNA was extracted from cultured cells or mice tissues with RNAiso Plus (TaKaRa, Cat. 9109). Genomic DNA removal and reverse transcription reactions were determined with the PrimeScript™ RT reagent Kit (Roche, Cat. RR037A) and Genomic DNA Eraser. cDNA samples were amplified by StepOne Plus Real-Time PCR System (Applied Biosystems) using SYBR Green PCR Master Mix. The target gene expression levels were normalized to GAPDH, and relative expression was determined using the Δ Ct relative Quantification method (14). The *in vitro* RT-PCR experiments were repeated three times. The primer sequences for RT-PCR were listed in **Table S2**.

### 2.8 Transcriptome sequencing

Mouse peritoneal macrophages were isolated and treated with vehicle or C498 (15 μM) for 0.5 h, followed by LPS (100 ng/ml) challenge for an additional 4 h (n = 2 per group). Total RNA was isolated using TRIzol (Thermo Fisher Scientific) and genomic DNA was removed using DNase I (Takara). RNA purification, reverse transcription, library construction, and sequencing were determined. The mRNA was fragmented and the short sequence fragments were sequenced using the Illumina platform. The final library was then sequenced on Illumina Nova Seq6000. The raw sequencing data were filtered with fastp. The clean data were then compared with the reference genome using the HISAT2 (20) software. The mapped reads for each sample were assembled by StringTie. The expression levels were quantified separately using the software RSEM. Differential expression analysis was determined using DESeq2, and genes with fold change (FC)≥ 2 and P-adjust ≤ 0.05 were considered as significantly differentially expressed genes. KEGG pathway enrichment analysis (21), PPI (22), GSEA (23), Reactome (24), trend (25) and IPA analysis (26) of DEGs were performed.

### 2.9 Mouse sepsis model

Mice were randomly divided into three groups (n = 6 per group) and intraperitoneally injected with either vehicle (10% HS-15 + 2% DMSO in PBS buffer) or 5 mg/kg of C498 dissolved in 10% HS-15 + 2% DMSO PBS buffer. Twelve hours later, mice were challenged i.p. with 6 mg/kg of LPS. The body temperature of mice was monitored. The *in vivo* mouse septic experiments were repeated three times.

### 2.10 HE staining

Mouse lung and kidney tissues embedded in paraffin were sectioned and stained with H&E according to a standard protocol (27). Briefly, paraffin-embedded mouse tissue sections were deparaffinized in xylene and rehydrated in serial decreasing concentrations of ethanol. The slides were then stained with Hematoxylin solution, Hematoxylin Differentiation solution and Hematoxylin Scott Tap Bluing sequentially. Next, the slides were dehydrated with 85% and 95% ethanol and finally stained with Eosin dye. Then, the sections were dehydrated successively in gradient ethanol and xylene, mounted with resin mounting medium and observed with microscope inspection.

### 2.11 Immunofluorescence staining

Paraffin-embedded tissue sections were deparaffinized in 2 changes of xylene and rehydrated in serial decreasing concentrations of ethanol (100%, 85% and 75%). The sections were then immersed in EDTA antigen retrieval buffer (pH 8.0) (Servicebio, G1206) and held at sub-boiling point temperature for 8 min twice. After washing three times with PBS (pH 7.4) in a Rocker device (Servicebio, TSY-B), the sections were further covered to block non-specific binding in 3% BSA for 30 min, followed by incubation overnight at 4°C in a wet box with primary antibody CD11b or IL-6 prepared in PBS at a certain ratio (1:500). After washing 3 times for 5 min each on a rocker in PBS (pH 7.4), sections were covered with fluorescent-CY3 secondary antibody (Servicebio, GB21303), and incubated for 50 min at room temperature in dark. After incubating with DAPI solution (Servicebio, G1012) at room temperature for 10 min in dark, sections were incubated with spontaneous fluorescence quenching reagent (Servicebio, G1221) for 5 min. Then, the sections were mounted with an anti-fade mounting medium (Servicebio, G1401). Microscopy detection and collection of images were performed by Fluorescent Microscopy (Nikon, NIKON ECLIPSE C1). The color channel of the fluorescence was adjusted using CaseViewer software. The quantification was determined by ImageJ software.

### 2.12 Target capture technology based on surface plasmon resonance

C498 were formulated with DMSO and immobilized on a 3D photo-crosslinked chip surface by array spotting using a BioDot™-1520 array printer. Peritoneal macrophage lysate was used as the liquid phase and a microfluidic environment was established. The binding of protein targets interacting with C498 on the surface of the chip was monitored in real-time by the surface plasmon resonance (SPR) assay. C498 Dotted area signal curve (red) indicates the signal change in the dotted area of the compound on the chip, while the background noise signal curve (black) indicates the signal change in the unsampled area (data not shown). After testing, the chips were subjected to *in situ* enzymatic digestion, followed by identification of the protein species enriched on the chip surface through HPLC-MS assay (TripleCore Tech China). A total of 144 proteins were analyzed and confirmed, including 89 high-score targets (> 1000), 50 mid-score targets (> 200 and < 1000) and 5 low-score targets (< 200). Bioinformatic analysis was performed to identify targets.

### 2.13 Molecular docking

The molecular structure files of the target proteins were downloaded from PDB database (https://www1.rcsb.org/). PyMOL 2.3.0 software was used to perform operations such as the deletion of water molecules and the deletion of proligands for the downloaded target proteins. C498 small molecule structure files were downloaded from PubChem database. Chem3D (ver. 2020) software was used to obtain the optimal conformation of the energy-minimized small molecule. Auto Dock Tools 1.5.6 was used to hydrogenate target protein molecules and determine the twistable bonds of small molecules. Protein activity pockets were predicted using the POCASA protein activity pocket online prediction tool. Molecular simulation docking of target proteins and small molecules was performed using Auto Dock Vina v.1.2.0 software and visualized using PyMOL 2.3.0 software and Discovery Studio 2020.

### 2.14 Statistical analysis

All the histograms and line charts were made by GraphPad Prism 9.0. Results were graphed as mean ± SEM. Significant differences between data groups were obtained by unpaired Student’s t-test or one-way or two-way ANOVA. Significant differences are indicated as * P-value < 0.05.

## 3 Results

### 3.1 C498 is identified as a dual-target inhibitor of JAK/STAT and NFκB signaling

**Figure 1:**
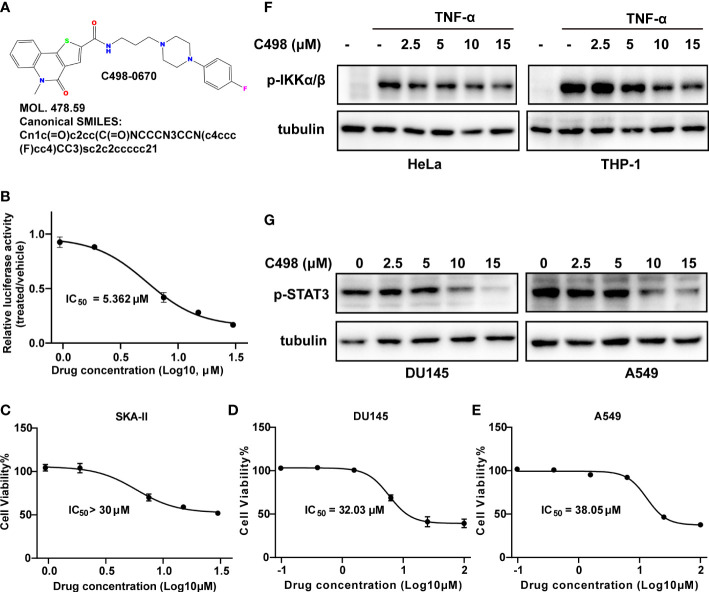
C498 is identified as a dual-target inhibitor of STAT and NFκB signaling pathways by a luciferase drug screening system. **(A)**, The chemical structure, molecular weight, and Canonical SMILES of C498. B-E, SKA-II, DU145 and A549 cells were seeded in 96-well plates and cultured overnight. Cells were treated with DMSO or a serial of C498 for 24 h. Luciferase activities of SKA-II cells were determined after 24 h **(B)**. The cell viability of SKA-II **(C)**, DU145 **(D)** and A549 **(E)** cells was determined after 24 h using resazurin assay. **(F)** HeLa and THP-1 cells were treated with DMSO or C498 at 2.5, 5, 10, and 15 μM for 2 h, followed by TNF-α stimulation (20 ng/ml, 10 min). Western blot analysis was determined on whole cell lysates and detected with anti-pSer176/180-IKKα/β antibodies. Tubulin was used as a loading control. **(G)** A549 and DU145 cells with constitutive STAT3 activation were treated with DMSO or C498 at 2.5, 5, 10, and 15 μM for 2 h. Western blot analysis was determined on whole cell lysates and detected with an anti-pTyr705-STAT3 antibody. Tubulin was used as a loading control.

As we previously described (14), we screened a total of 18,840 small molecules through a high-throughput drug screening workflow (**Figure S1A**). Those compounds with inhibitory activities were further filtered by searching the PubMed database to omit previously reported compounds that interacted directly or indirectly with JAK/STAT and NFκB signaling. After that, C498-0670 (C498) from TargetMol Bioactive Compounds Library Plus (Cat. D7800) (**Figure 1A**), for which no biological activity reports were retrieved in PubMed, was identified as one of the most potent inhibitors for dual JAK/STAT and NFκB luciferase activities. The C498 drug-likeness prediction by SwissADME tool (28) shows that C498 completely obeys the Lipinski rule of five (29) (below 5 hydrogen bond donors, 10 hydrogen bond acceptors, 10 rotatable bonds, MLOGP < 4.15 and 500 molecular weight) with 0 violations, confirming its drug-like capability. Next, the inhibition of luciferase activity and cytotoxicity were explored in a serial of doses. As shown in **Figure 1B**, C498 effectively inhibited luciferase activity in a dose-dependent manner (IC_50_ = 5.362 µM). The IC_50_s for growth inhibition of luciferase based cell line SKA-II (14) and immortalized cell lines A549 and DU145 were more than 30 µM (much higher than luciferase inhibitory IC_50_ value), pointing to its low cytotoxicity (**Figures 1C-E**). To distinguish whether C498 inhibits STATs, NFκB, or both, we examined the influence of C498 on these two signaling pathways separately. As shown in **Figure 1F**, C498 inhibited TNF-α-induced phosphorylation of IKKα/β in a dose-dependent manner in the immortalized cell lines HeLa and THP-1 cells, indicating its inhibitory activity on NFκB signaling. Besides, C498 also inhibited the constitutive activation of STAT3 in A549 and DU145 cells (**Figure 1G**), indicating its inhibitory activity on STAT signaling. These data suggest that C498 has a dual inhibitory effect on JAK/STAT and NFκB signaling pathways.

### 3.2 C498 inhibits JAK/STAT and NFκB signaling and downstream gene expression in mouse primary peritoneal macrophages

**Figure 2:**
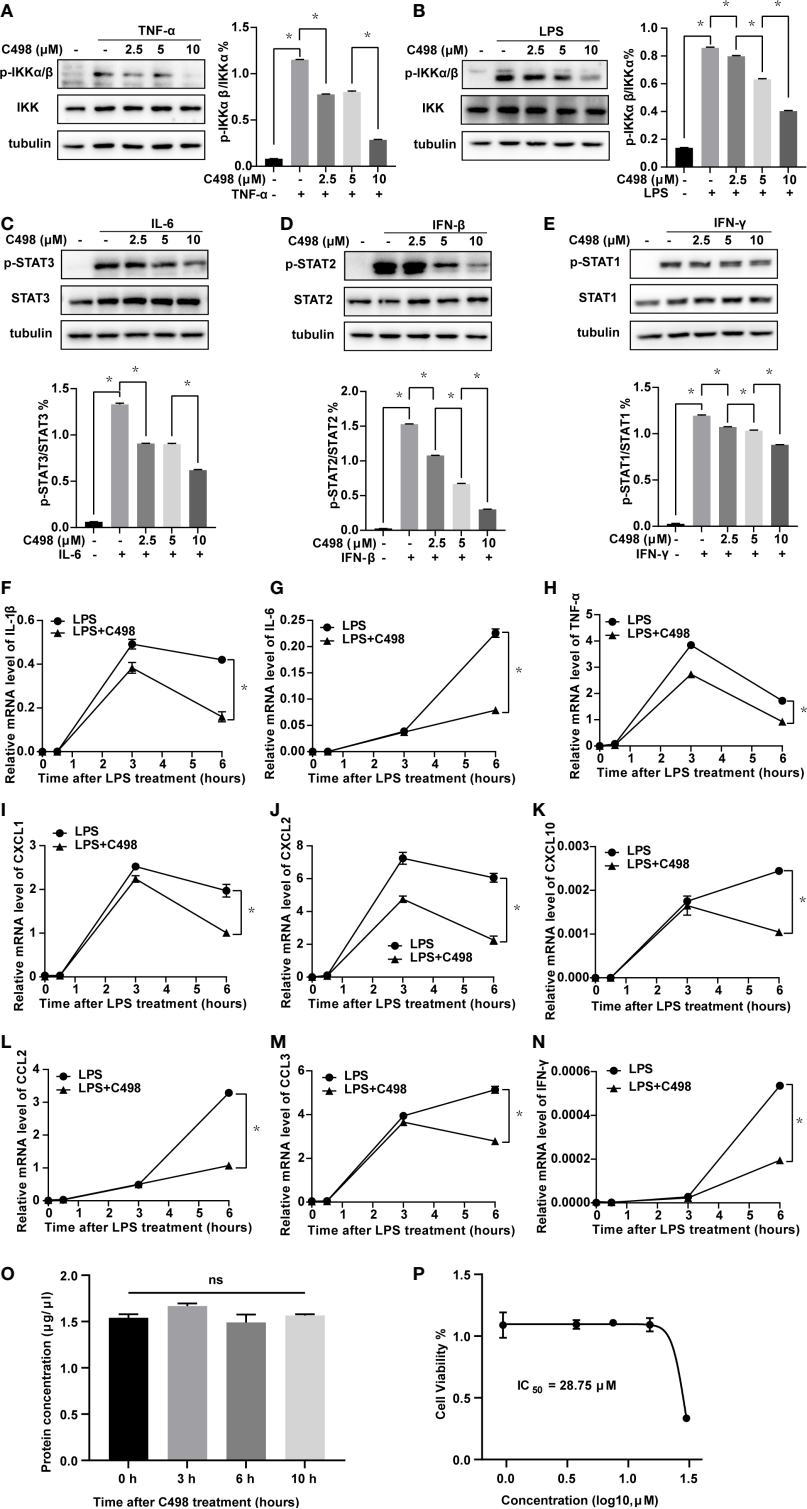
C498 inhibits JAK/STAT and NFκB signaling pathways and downstream gene expression in mouse peritoneal macrophages. **(A-E)** Peritoneal macrophages were isolated and treated with DMSO or C498 at 2.5, 5, and 10 μM for 2 h, followed by stimulation with 20 ng/ml TNF-α for 10 min or 100 ng/ml LPS for 0.5 h (**A**, **B**), 20 ng/ml IL-6 **(C)**, 50 ng/ml IFN-β and IFN-γ (**D**, **E**) for 10 min. Western blot analysis of whole cell lysates and quantitative analysis were determined with primary antibodies including anti-pSer176/180-IKKα/β and anti-IKK (**A**, **B**), anti-pTyr705-STAT3 and anti-STAT3 **(C)**, anti-pTyr690-STAT2 and anti-STAT2 **(D)**, anti-pTyr701-STAT1 and anti-STAT1 **(E)**. F-N, Peritoneal macrophages were pretreated with DMSO or C498 (5 μM) for 30 min and then stimulated with 150 ng/ml LPS for additional 0, 0.5, 3, and 6 h. The relative mRNA levels of IL-1β **(F)**, IL-6 **(G)**, TNF-α **(H)**, CXCL1 **(I)**, CXCL2 **(J)**, CXCL10 **(K)**, CCL2 **(L)**, CCL3 **(M)** and IFN-γ **(N)** were determined by RT-PCR. **(O)** Peritoneal macrophages were treated with 5 μM C498 for 0, 3, 6 and 10 h, cell lysates were harvested, and protein concentrations were determined. **(P)** Peritoneal macrophages were seeded in 96-well plates, cultured overnight, and treated with DMSO or a serial of C498. Cell viability was determined after 24 h. *P < 0.05 is considered significant. ns, not significant.

Considering the essential role of JAK/STAT and NFκB signaling pathways in acute inflammatory diseases and immune cell responses (11), we further investigated the inhibitory effects of C498 on innate immune cells, such as primary peritoneal macrophages. Cytokines (IL-6, IFN-γ, and IFN-β) induced JAK/STAT signaling, and LPS and TNF-α induced NFκB signaling, are both engaged in macrophage-driven immune responses (30). Consistent with the findings in **Figure 1**, C498 effectively inhibited TNF-α and LPS-induced IKKα/β phosphorylation (**Figures 2A, B**), IL-6-induced STAT3 activation (**Figure 2C**), and IFN-β induced STAT2 activation and IFN-γ-induced STAT1 phosphorylation (**Figures 2D, E**). Since LPS can sequentially activate JAK/STAT and NFκB signaling, we investigated the effects of C498 on the expression of major downstream genes under LPS stimulation. C498 diminished LPS induced gene expressions such as IL-1β, IL-6, TNF-α, CXCL1, CXCL2, CXCL10, CCL2, CCL3, and IFN-γ (**Figures 2F-N**). The protein concentrations could roughly indicate the cell number, which were not altered by C498 treatment at 5 μM for up to 10 h (**Figure 2O**). The IC_50_ value of cell viability for mouse primary peritoneal macrophages was 28.75 μM, which is much higher than the concentration for signaling inhibition in **Figures 2A-N** (**Figure 2P**). These data suggest that C498 exhibited anti-inflammatory effects by inhibiting the JAK/STAT and NFκB pathways and their downstream gene expression in primary macrophages *in vitro* with no observed cytotoxicity.

### 3.3 Transcriptome profile systematically illuminates the anti-inflammatory effects of C498 in peritoneal macrophages

**Figure 3:**
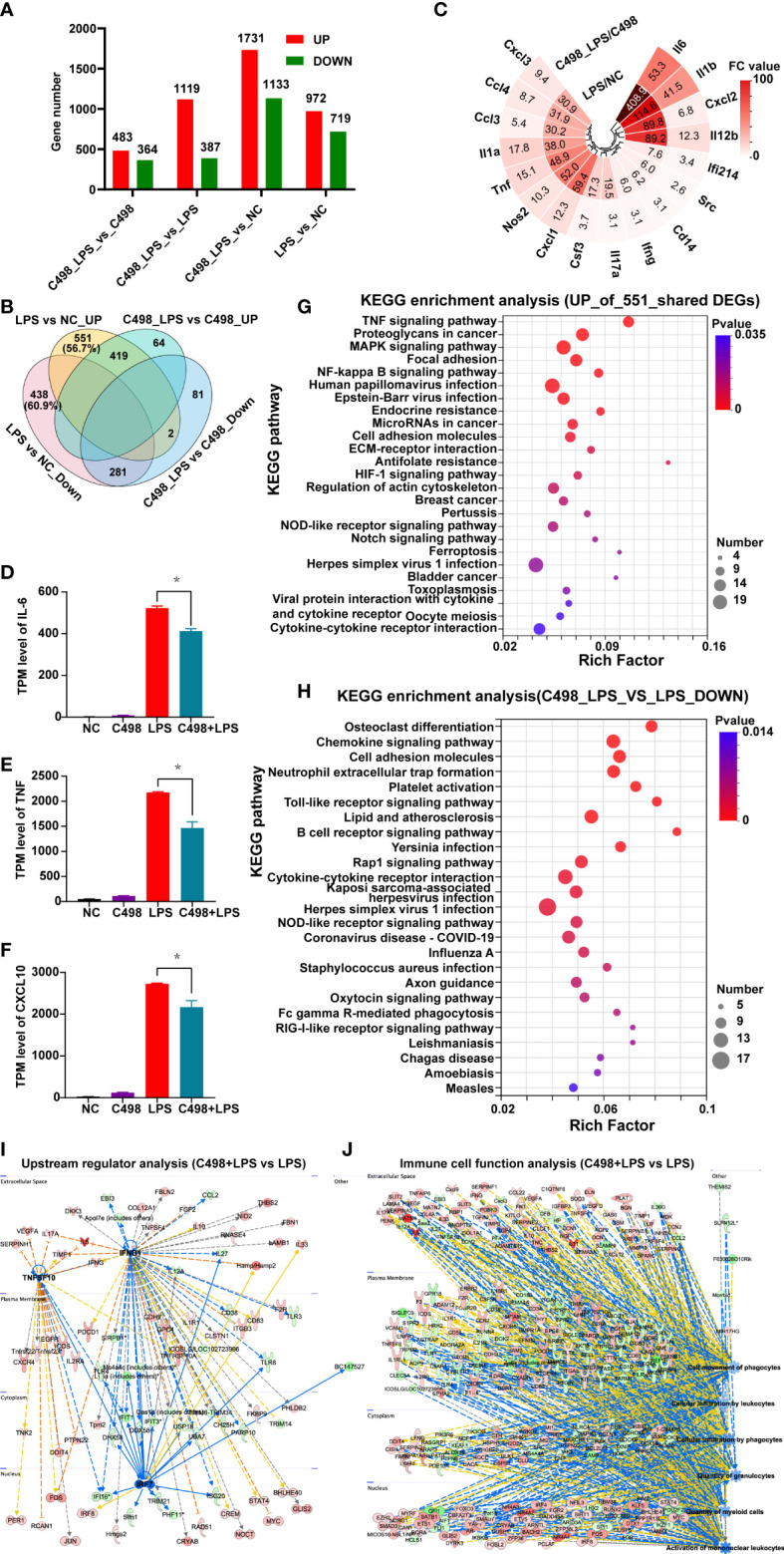
Anti-inflammatory effects of C498. **(A)**, DEGs among different treatment groups. Genes with fold change (FC) ≥ 2 and P-adjust ≤ 0.05 were considered as significantly differentially expressed genes. **(B)** Venn diagram of DEGs among indicated comparison groups. **(C)** Fold change values (FC) of canonical inflammatory factors enriched in 551 DEGs in **(B)**. D-F, The normalized expression (TPM levels) of IL-6 **(D)**, TNF **(E)** and CXCL10 **(F)** was graphed. *P < 0.05 is considered significant. **(G)**, The top 25 KEGG enrichment analysis of the upregulated 511 DEGs in **(B)**. **(H)**, The top 25 of KEGG enrichment of downregulated DEGs between C498_LPS vs LPS groups. **(I)**, The decreased upstream regulators IRF7-IFNB-TNFSF10 related networks of C498_LPS vs LPS in IPA analysis. **(J)**, The decreased immune cell trafficking functions of C498_LPS vs LPS in IPA analysis.

Following our investigation of C498 on immune cells, we performed transcriptome analysis to thoroughly elucidate the anti-inflammatory effects of C498. As shown in **Figure S1B**, peritoneal macrophages treated with vehicle (NC), LPS or C498 alone, or combination of C498 and LPS were collected and processed for RNA sequencing (GSE220654). As expected, LPS treatment significantly regulated numerous gene expression changes (**Figure 3A** and **S2A**) (14, 31). KEGG functional enrichment analysis of the upregulated DEGs identified various upregulated proinflammatory signaling pathways, such as TNF signaling pathway, NFκB signaling pathway, Toll-like receptor signaling pathway, and NOD-like receptor signaling pathway (**Figure S2B**). Similar proinflammatory pathways were also observed in GSEA analysis (**Figure S2C-F**). Using IPA analysis, downstream mediators of LPS binding receptor TLR4, including TNF, IL-1β, IL-6, IFN-γ, NFκB, and STAT3 were predicted to be activated by LPS (**Figure S2G-H**). TNF, NOD-like receptor signaling pathway, and NFκB signaling-related leukocyte activation and shock response are both enriched in the LPS upregulated networks (**Figure S2G-H**). In the PPI network, IL-1, IL-6, TNF, CXCL1, CXCL2, and JAK2 are identified as hub genes to regulate responses to LPS treatment (**Figure S2I**). All of these data verify the pivotal roles of JAK/STAT and NFκB signaling in LPS-induced inflammatory responses in macrophages.

Next, LPS induced gene expression changes were examined in C498 treated groups to explore its influence on immune responses post LPS challenge. As shown in **Figure 3A**, there were 483 upregulated DEGs and 364 downregulated DEGs of C489+LPS vs C498 when compared to the 972 upregulated and 719 downregulated DEGs of LPS vs NC. Besides, Venn analysis of DEGs among indicated comparison groups demonstrated that more than half of DEGs post LPS challenge was no longer significantly upregulated (551 out of 972 (56.7%)) or downregulated (438 out of 719 (60.9%)) under C498 pretreatment (**Figure 3B**). Especially, a lot of canonical inflammatory factors post LPS challenges (14) were enriched in these 551 DEGs (**Figure 3C**). Consistent with RT-PCR data in **Figure 2**, the expression levels of IL-6, TNF, and CXCL10 were significantly inhibited in C498+LPS vs LPS groups according to our RNA-seq data (**Figures 3D-F**). Next, KEGG functional enrichment analysis was performed on these 551 and 438 genes to explore the influence of C498 on LPS induced gene expression changes (**Figure 3G**, **S3A**). Proinflammatory functional terms such as TNF signaling pathway, NFκB signaling pathway, and NOD-like receptor signaling pathway are enriched in the top 25 KEGG pathways of the 551 genes (**Figure 3G**). A panel of metabolic signaling pathways and immunosuppressive PPAR signaling pathway were enriched in 438 DEGs downregulated by LPS but not by C498+LPS (**Figure S3A**). Trend analysis also identified a panel of genes that were upregulated in LPS but reversed post C498+LPS treatment (Profile 5), and a panel of genes which were downregulated in LPS but recovered post C498+LPS treatment (Profile 2) (**Figure S4A, B**). KEGG functional enrichment analysis also confirmed the anti-inflammatory effects of C498 in LPS treated peritoneal macrophages (**Figures S4C, D**).

In addition, by comparing C498+LPS with LPS groups, there are 1119 significantly upregulated genes and 387 significantly downregulated genes between C498+LPS and LPS groups (**Figure 3A**). KEGG analysis of downregulated DEGs indicates that signaling pathways such as Cell adhesion molecules, Chemokine signaling pathway, Toll-like receptor signaling pathway and NOD-like receptor signaling pathway were downregulated by C498+LPS compared with LPS samples (**Figure 3H**). The anti-inflammatory TGF-beta signaling pathway was upregulated by C498+LPS when compared to LPS samples (**Figure S3B**). Consistently, immunosuppressive upstream regulator IL-10RA was activated (**Figure S3C**) while canonical proinflammatory TNFSF10, IFNB1 and IRF7 were inhibited by C498+LPS when compared to the LPS group (**Figure 3I**). Immune cell trafficking processes (**Figure 3J**) including cellular infiltration by phagocytes, cellular infiltration by leukocytes and activation of mononuclear leukocytes, as well as inflammatory diseases, such as rheumatoid arthritis, lung inflammation, dermatitis, and immune-mediated inflammation disease, are predicted to be suppressed by C498+LPS vs LPS group (**Figure S3D**).

These data suggest that C498 treatment did interfere with LPS-induced gene expression profiles and proinflammatory functional responses.

### 3.4 Comparative analysis of C498 anti-inflammatory efficiency using IPA

**Figure 4:**
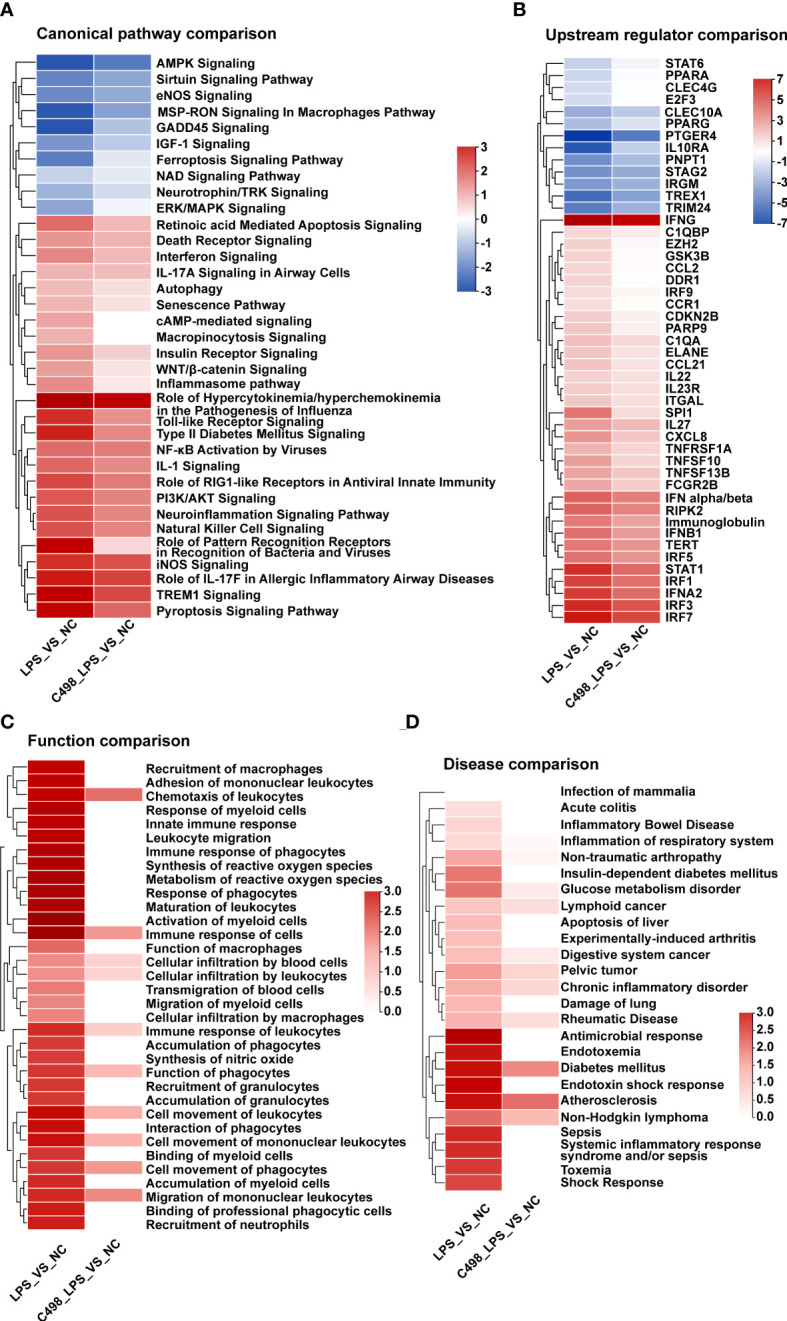
Comparative analysis of C498 anti-inflammatory efficiency. **(A-D)**, Canonical Pathway Comparison **(A)**, Upstream Regulator Comparison **(B)**, Biological Function Comparison **(C)**, and Disease Comparison **(D)** were conducted by comparing the significant differences between (C498_LPS vs NC) and (LPS vs NC). Positive z scores indicate an increase whereas negative ones indicate a reduction in specific terms.

To further elaborate the anti-inflammatory effects of C498, we performed comparison analysis between LPS vs NC and C498+LPS vs NC using IPA software (**Figures 4A-D**). In canonical pathway comparison (**Figure 4A**), the signaling strength of various signaling pathways upregulated by LPS is generally diminished by C498 treatment (lower signaling strength in C498+LPS vs NC), especially for proinflammatory signaling pathways, such as Toll-like receptor signaling, inflammasome signaling, and role of Pattern Recognition Receptors in recognition of bacteria and viruses. In upstream regulator comparison (**Figure 4B**), activities of upregulated upstream regulators, including CCL2, IRF3/7/9, CXCL8, and IFN-α/β, were higher in LPS vs NC than C498+LPS vs NC. Meanwhile, C498 hindered the decreased activities of anti-inflammatory upstream regulators 10 (IL-10RA), PPARA, PPARG, and STAT6 post LPS treatment. In biological function comparison, multiple functions dramatically increased by LPS such as innate immune response, recruitment, accumulation, and the activation of mononuclear leukocytes, myeloid cells, and macrophages were retarded in C498+LPS vs NC comparison (**Figure 4C**). For disease comparison (**Figure 4D**), the majority of terms hindered by C498 treatment are related to inflammation diseases such as sepsis, shock response, systemic inflammatory response, damage of lung, rheumatic disease, acute colitis, and inflammation of respiratory system.

The comparison analysis between LPS vs NC and C498+LPS vs LPS was also performed (**Figure S5**). Canonical pathways such as TREM1 signaling were induced by LPS but inhibited by C498, while pathways like TGF-β signaling were decreased by LPS but activated by C498 (**Figure S5A**). Consistently, upstream regulator IL-10RA downregulated by LPS was activated by C498, and regulators like IFNB1 and STAT1 upregulated by LPS were inhibited by C498 (**Figure S5B**). The immune cell activation and inflammatory diseases caused by LPS were alleviated by C498 treatment (**Figure S5C**).

All of these data confirm that C498 exerts anti-inflammatory effects, indicating its potential applications in inflammatory diseases like sepsis.

### 3.5 C498 treatment ameliorates LPS-induced septic shock *in vivo*

**Figure 5:**
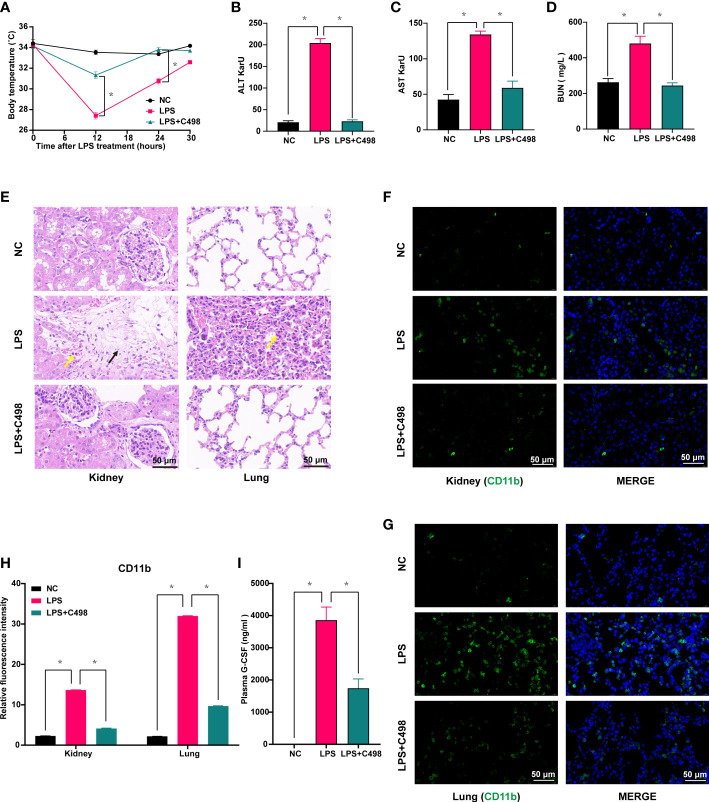
C498 prevents temperature loss and tissue damage caused by LPS-induced septic shock in vivo. Mice were i.p. administrated with 5 mg/kg C498 12 h before the LPS challenge (6 mg/kg, i.p.). Body temperature **(A)** was recorded. Mice treated as in **(A)** were sacrificed 24 h after LPS injection and plasma ALT **(B)**, AST **(C)**, and BUN levels **(D)** were measured. Mice treated as in **(A)** were sacrificed 12 h after LPS injection and HE staining (100 ×) of inflammatory cell infiltration in the lung and kidney **(E)**. Yellow arrows indicate neutrophil infiltration and black arrows indicate nuclei fragmentation. Mice treated as in **(A)** were sacrificed 12 h after LPS injection. Immunofluorescence **(F, G)** and relative fluorescence intensity (100 ×) of kidney and lung **(H)**. The green color indicates CD11b staining and blue indicates nuclear DAPI staining **(F-H)**. **(I)** The detection of 24 h plasma G-CSF levels by ELISA assay. *P < 0.05 is considered significant.

**Figure 6:**
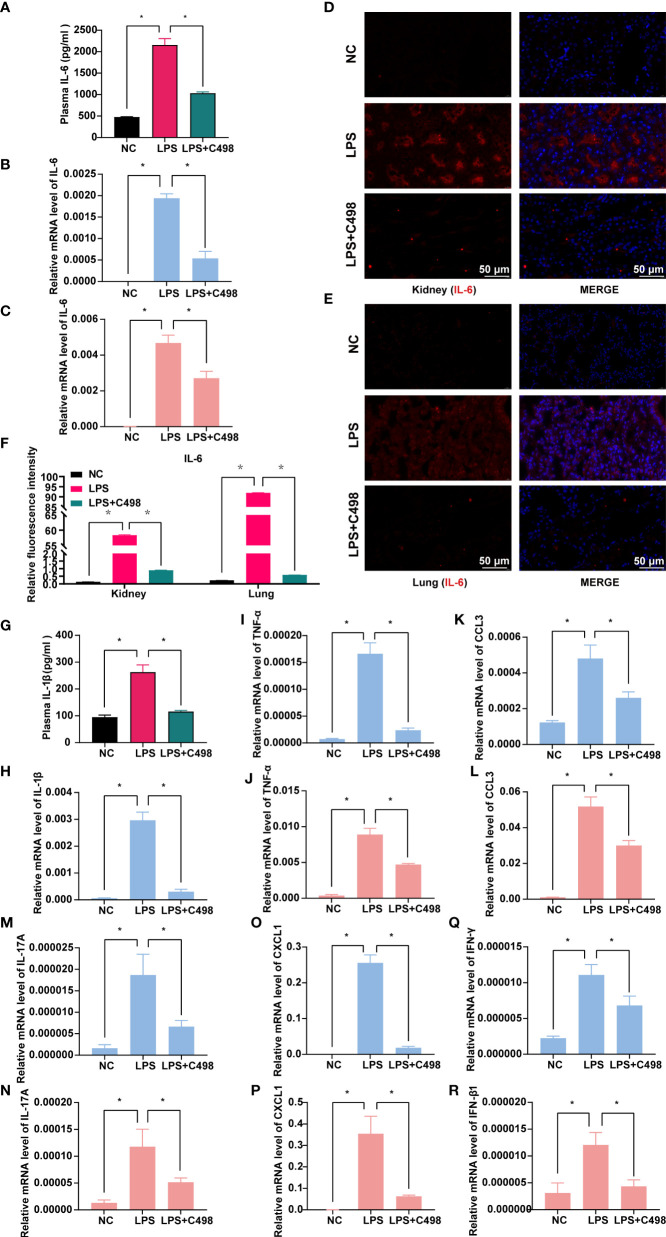
C498 inhibits the expression of relevant inflammatory factors in sepsis models. Mice were i.p. administrated with 5 mg/kg C498 12 h before the LPS challenge (6 mg/kg, i.p.) and sacrificed at 12h or 24h after LPS injection. The detection of 24 h plasma IL-6 levels by ELISA assay **(A)**. Mice treated as in **(A)** were sacrificed 12 h after LPS injection and the relative mRNA levels of IL-6 in kidney **(B)** and lung **(C)** were determined by RT- PCR. Immunofluorescence **(D, E)** and relative fluorescence intensity **(F)** of kidney and lung (100 ×). Red color indicates IL-6 staining and blue indicates nuclear DAPI staining **(D-F)**. The detection of 12 h plasma IL-1β levels **(G)** by ELISA assay and the relative mRNA levels of IL-1β in kidney **(H)** by RT- PCR. The mRNA levels of TNF-α **(I, J)**, CCL3 **(K, L)**, IL-17A **(M, N)**, CXCL1 **(O, P)**, IFN-γ **(Q)**, and IFN-β **(R)** within kidney **(I, K, M, O, Q)** and lung **(J, L, N, P, R)** tissues were determined by RT- PCR. *P < 0.05 is considered significant.

LPS, the major cell wall component of Gram-negative bacteria, induces severe systemic inflammatory responses *in vivo*, while an LPS-induced mouse septic shock model mimics the pathological process of human sepsis (32). We examined whether C498 did ameliorate the LPS-induced sepsis *in vivo*. As shown in **Figure 5A**, LPS injection caused a dramatic loss of body temperature, which was significantly hindered by C498 treatment. Tissue damage such as liver failure and kidney injury are considered major complications of septic shock (33). Plasma levels of ALT and AST to indicate liver damage and BUN for kidney damage were increased in LPS challenged mice and entirely or almost went back to normal post C498 treatment (**Figures 5B-D**). The pathogenic diagnosis of kidney and lung tissues by HE staining indicated that LPS stimulates neutrophil infiltration in lung and kidney tissue (yellow arrow) (**Figure 5E**). Nuclei fragmentation was also observed, which indicates tubular destruction (black arrow) in LPS treated kidney tissues, which was not noticed in LPS+C498 group. Accordingly, the infiltration of CD11b+ myeloid cells (including neutrophils) was increased in kidney and lung tissues post LPS injection and C498 reversed this trend (**Figures 5F-H**). The plasma level of granulocyte colony-stimulating factor (G-CSF), the growth factor for neutrophil activation in the early stages of sepsis (34), was also increased post LPS challenge and inhibited in C498+LPS group (**Figure 5I**). In consistency with the observed inhibition of proinflammatory cytokine and chemokine production by C498 *in vitro* (**Figures 2F–N**), numerous proinflammatory factors were induced in kidney and lung tissues by LPS injection, which were impaired by C498 treatment (**Figure 6**). For example, the systemic plasma IL-6 level (**Figure 6A**), and kidney and lung tissues’ IL-6 mRNA level (**Figures 6B, C**) and protein level (**Figures 6D-F**) were all increased in LPS group, but inhibited in C498+LPS mice. Both systemic plasma IL-1β protein level and mRNA level of kidney tissues were induced by LPS but diminished by C498 treatment (**Figures 6G, H**). Additionally, mRNA levels of TNF-α, CCL3, CXCL1, IL-17A and other inflammatory factors within kidney and lung tissues also exhibited the similar trend of changes (**Figures 6I-R**). We also evaluated the potential toxicity of C498 *in vivo* (**Figure S6**). C498 treatment at 5 and 10 mg/kg did not significantly change mouse body weight (**Figure S6A**), as well as weights of spleen (**Figure S6B**), liver (**Figure S6C**) and kidney (**Figure S6D**). There were also no observed changes of plasma levels of AST and BUN (**Figure S6E, F**). These results showed that C498 alleviates the inflammatory status within local tissues caused by LPS stimulation. Overall, these findings proved the protective role of C498 in LPS-induced septic shock and the low-toxicity for *in vivo* administration.

### 3.6 Target fishing of C498 compound

**Figure 7:**
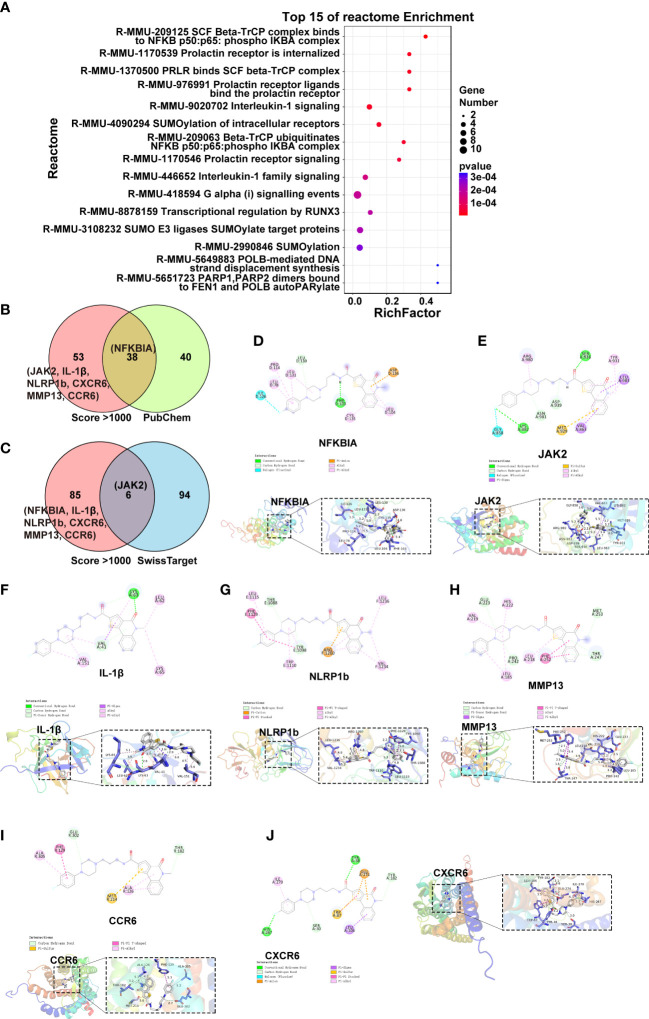
C498 putative drug targets. **(A)**, The Reactome analysis of 89 targets (score > 1000) of C498. **(B)**, Venn analysis of PubChem Biological Test and target fishing results. **(C)**, Venn analysis of target fishing results and SwissTarget predicted targets. **(D-J)**, The docking results were made separately in 2D and 3D plots using Pymol (2.3) and Discovery Studio2020.

To better understand its mechanism of action (MOA), we performed target fishing of C498 using the SPR,HPLC and MALDI-TOF-MS approach (35). A total of 144 targets were identified and 89 targets exhibited high affinities (score > 1000). Reactome enrichment analysis of these 89 targets showed that the targets are related to phospho IKBA complex, IL-1 signaling, and IL-1 family signaling (**Figure 7A**). Among them, the putative target NF-kappa-B inhibitor alpha (NFKBIA, score 1600.89) inhibits the activity of NFκB/REL complexes to interfere with NFκB signaling while IL-1β (score 1445.32), NLR family, and pyrin domain containing 1b (NLRP1b, score 1121.29) disturb IL-1β signaling pathways. Additionally, the putative target JAK2 (score 1336.33) is the key kinase mediator for STAT activation which might be responsible for the JAK/STAT inhibition by C498. As a bioactive compound, a panel of putative targets of C498 was identified using cell based qHTS assay in the Biological Test Results section of PubChem (PubChem CID. 16020277) (36). We compared those 78 drug targets in PubChem with the 89 drug targets with high binding affinity (**Figure 7B**). The drug target NFKBIA was also verified in the PubChem database while IL-1β, NLRP1b and JAK2 were the novel targets, which have never been reported. We also did target prediction using the SwissTarget database (37), which verified 6 drug targets including JAK2 (**Figure 7C**). In addition to these 4 targets, there were other 3 inflammation-related targets with high affinities (CXCR6, MMP13 and CCR6). To further verify the targets, we performed simulations of binding activity and sites using a computer-based molecular docking analysis (38). As shown in **Table 1**, the binding energy (vina score < -7kcal/mol) of these targets indicates excellent docking of small molecules with proteins. The molecular docking model shows that the binding site of C498 on NFKBIA consists of the following residues: amino acid residues LEU-130, PRO-114, LEU-78/104/131, CYS-135, ASP-136, PHE-103, and ILE-126 in the D-chain of the protein (**Figure 7D**). The binding sites of C498 on JAK2 include GLY-858, LYS-882, ASN-981, ASP-939, MET-929, VAL-863, and LEU-983 in the A-chain (**Figure 7E**). C498 also binds strongly to the IL-1β and NLRP1b proteins (**Figures 7F, G**), demonstrating the target-based inhibitory effects of C498 on IL-1β signaling. Moreover, inflammation-related targets (CXCR6, MMP13, and CCR6) are all closely related to the progression of the inflammatory response, and C498 shows a strong bonding effect with all of them (**Figures 7H-J**). In summary, by target fishing, database matching and molecular docking, we identified putative targets JAK2 for JAK/STAT signaling, NFKBIA for NFκB signaling, and IL-1β and NLRP1 for IL-1 signaling.

**Table 1:** Molecular docking results of inflammatory related C498 drug targets.

Target name	Vina Score
MMP13	-11
JAK2	-10.3
CXCR6	-9.2
NLRP1b	-9.1
CCR6	-8.4
IL-1β	-7.7
NFKBIA	-7.6

## 4 Discussion

The excessive inflammatory response can cause tissue damage and abnormal organ function, severe systemic symptoms, and human mortality. Sepsis, a syndrome of multiple organs or tissue damage resulting from a systemic inflammatory response caused by infection and trauma, is a major global health concern and the leading cause of in-hospital death in the world (39). JAK/STAT and NFκB pathways are critical in the regulation of inflammation. The two pathways often co-occur, share common upstream stimulators and downstream released proinflammatory factors, and commit frequent crosstalk and physical interactions. Therefore, for drug development, it is a promising option to target both JAK/STAT and NFκB signaling *via* drug combination or dual-target drugs. In this study, we conducted a high-throughput luciferase-based drug screening project targeting the dual signaling response of JAK/STAT and NFκB activity (14), and identified the novel bioactive compound C498 as a dual inhibitor of JAK/STAT and NFκB signaling pathways. Using *in vitro* experiments combined with transcriptomic analysis, C498 is proven to inhibit key protein activities of JAK/STAT and NFκB pathways and downstream proinflammatory gene expression. The anti-inflammatory efficacy of C498 is also demonstrated in an *in vivo* sepsis model. Furthermore, we identified putative targets JAK2, NFKBIA, NLRP1b and IL-1β by target fishing, database searching, and molecular docking, which could be responsible for its anti-inflammatory effects. All of these data suggest that C498 is a promising anti-inflammatory drug for the treatment of various inflammatory diseases.

Traditional therapeutic options for inflammation include the use of glucocorticoids (GCs) and non-steroidal anti-inflammatory drugs (NSAIDs). However, the use of classical anti-inflammatory therapies with NSAIDs and cyclooxygenase 1/2 (COX1/2) inhibitors can cause a variety of side effects, such as osteoporosis, infections, digestive tract injury, and increased risk of thrombosis (40). Researchers are therefore focusing on the development of next-generation anti-inflammatory agents that are different from NSAIDs and COX1/2 inhibitors. For example, tofacitinib, a JAK3-specific inhibitor, is used to treat ankylosing spondylitis (AS), rheumatoid arthritis (RA), psoriasis, psoriatic arthritis, ulcerative colitis, polyarticular juvenile idiopathic arthritis, etc. (41). Similarly, the JAK1 inhibitors Filgotinib and Upadacitinib are used to treat inflammatory bowel disease (42). STAT3 is a potential target for drug therapy (12), and several candidate compounds have already entered clinical studies (43, 44). Some small molecule NFκB inhibitors, such as pyrrolidine dithiocarbamate ammonium (PDTC), are also currently being investigated for the treatment of various inflammatory diseases, such as acute pancreatitis-like illness (13), cardiac inflammation (45), and rheumatoid arthritis. Given the close interaction of these two signaling pathways and the fact that inhibitors targeting both pathways exhibit similar anti-inflammatory applications (46), drugs with dual inhibitory activities may be more effective in the treatment of inflammatory diseases, such as septic shock.

In addition to the sepsis model used in this study, the potential therapeutic applications of C498 for other inflammatory diseases were also provided based on the disease/function analysis of our RNA-seq data. According to IPA disease comparison analysis, C498 alleviated a variety of inflammatory diseases including shock response, systemic inflammatory response, lung injury, rheumatic diseases, acute colitis, inflammatory bowel disease, and inflammation of respiratory system, not to mention the pivotal roles of JAK/STAT and NFκB signaling pathways in these inflammatory diseases. Besides, the putative drug targets of C498 *via* target fishing approach include numerous inflammation associated targets, not only JAK/STAT and NFκB related (JAK2 and NFKBIA), but also other inflammatory signaling pathways (IL-1β and NLRP1b for IL-1 signaling) and immune cell receptors/ligands (CXCR6, MMP13, and CCR6). There are also other targets indirectly related to inflammatory pathways, some of which have been validated in PubChem, such as MTOR, NFE2L2, CUL1, and SMAD3. However, these putative drug targets need to be validated through kinase assays, overexpression/knockdown experiments, and other approaches, respectively in our future work. Especially, the binding sites predicted by molecular docking need to be further explored using tools like gene point-mutation based kinase assay, SPR binding experiment, and cell-based activity determination and compound structural modification. The drug applications of C498 for other inflammatory diseases would also be validated.

As a bioactive compound, C498 exhibited not only anti-inflammatory effects, but also therapeutic potentials for metabolic diseases and certain cancers (**Figure 4D**) according to the RNA-seq data. The non-anti-inflammatory applications of C498 need to be further verified, as well as the connections of the identified targets with these treatment usages. In another way, as a multi-target drug, the therapeutic activities of C498 for specific disease conditions might be interfered by the targets with irrelevant or opposite functions (such as anti-inflammatory vs anti-tumour). Therefore, further structural modifications could be made based on the backbone portion of the C498 compound to improve the specific biological activity while reducing adverse side effects, even though C498 fully complies with the Lipinski rule of five.

In conclusion, we found C498 to be a novel anti-inflammatory compound targeting JAK/STAT, NFκB, and other inflammatory signaling pathways. The development of multi-target anti-inflammatory drug is a growing trend, and C498 has great potential for further therapeutic applications.

## Data availability statement

The datasets presented in this study can be found in online repositories. The names of the repository/repositories and accession number(s) can be found below: https://www.ncbi.nlm.nih.gov/geo/, GSE220654.

## Ethics statement

The animal study was reviewed and approved by Committee of Experimental Animals of the School of Medicine and Pharmacy, Ocean University of China (OUCSMP-20220301).

## Author contributions

QS and XL conceived and designed the experiments. JX, XZ, YX, LW, QZ, and ZW performed the experiments. JX and QS analyzed the data and wrote the paper. MZ, PL, XX, and QW polished the paper. PS provided the materials. All authors contributed to the article and approved the submitted version.
