# Supplementary material for: Bioactive compound C498-0670 alleviates LPS-induced sepsis via JAK/STAT and NFκB signaling pathways
Source: Front Immunol. 2023 Apr 14;14:1132265. doi: 10.3389/fimmu.2023.1132265 (PMC10140310; doi:10.3389/fimmu.2023.1132265)
Supplement: Supplementary file 1 [file DataSheet_1.docx]

Supplementary Material

Bioactive compound C498-0670 alleviates LPS-induced sepsis via JAK/STAT and NFκB signaling pathways

Jing Xu, Xiaoyu Li*, Qiaoling Song*

*** Correspondence:** Corresponding Author:

Xiaoyu Li, [lixiaoyu05@163.com](mailto:lixiaoyu05@163.com); Qiaoling Song, [sql_simm@163.com](mailto:sql_simm@163.com)

# Supplementary Figures and Tables

## Supplementary Figures

**FIGURE S1. Workflow for high-throughput drug screening and transcriptome analysis of C498**

A, Workflow of the high-throughput drug screening procedure. B, Workflow of sequencing samples. Peritoneal macrophages were isolated and treated with vehicle or C498 for 0.5 h, followed by LPS challenge for additional 4 h. Samples were collected and processed for RNA-seq analysis.

**FIGURE S2. LPS induces proinflammatory response in peritoneal macrophages.**

A, Hierarchical cluster analysis of DEGs between LPS and NC groups. The upregulated DEGs are indicated in red, and the downregulated DEGs are in blue. B, The top 25 category terms of KEGG analysis of upregulated DEGs between LPS and NC groups. C-F, GSEA data sets enriched in LPS upregulated gene clusters. The upstream regulator network (G) and graphical summary of significant changes (H) between LPS and NC group by IPA analysis. The increase or activation of target proteins, typical signaling, immune cell regulation, and symbolic prediction of disease are indicated in orange. I, PPI analysis of DEGs in LPS-treated group vs NC group. The blue symbols indicate downregulated DEGs while the red ones indicate upregulated DEGs. Circle size represents interaction degree and color strength represents values of log (FC).

**FIGURE S3. C498 exerts anti-inflammatory effects via RNA-seq analysis.**

A, The top 25 KEGG enrichment analysis of the downregulated 438 DEGs in Fig 3B. B, The top 25 of KEGG enrichment of upregulated DEGs between C498_LPS vs LPS groups. C, The increased upstream regulator IL10RA-related networks of C498_LPS vs LPS in IPA analysis. D, The inhibited inflammatory diseases of C498_LPS vs LPS in IPA analysis.

**FIGURE S4. Trend analysis among NC, LPS, and C498+LPS.**

Trend analysis was determined for all three groups of NC, LPS, and C498+LPS genes with non-zero expression, and profile 5 (A) and profile 2 (B) were obtained. (C) The top KEGG enrichment analysis of profile 5. (D) The top KEGG enrichment analysis of profile 2.

**FIGURE S5. Comparison analysis of NC vs LPS and LPS vs LPS+C498.**

The comparison analysis between LPS vs NC and C498+LPS vs LPS was performed. The representative canonical pathways (A), upstream regulator (B), and disease/function (C) were exhibited.

**FIGURE S6. Evaluation of C498 toxicity *in vivo*.**

Mice were i.p. administrated with 5 mg/kg or 10 mg/kg C498. Body weight (A) was recorded. Mice were sacrificed at 36 h and the weights for spleen (B), liver (C), and kidney (D), and plasma AST (E) and BUN levels (F) were measured.

## Supplementary Tables

**Table S1：List of Antibodies**

| **Antibody name** | **Catalog No.** | **Company** | **Dilution** |
| --- | --- | --- | --- |
| Anti-pTyr701-STAT1 | 9167 | Cell Signaling Technology | 1:1000 |
| Anti-STAT1 | 14994 | Cell Signaling Technology | 1:1000 |
| Anti-pTyr705-STAT3 | 9145 | Cell Signaling Technology | 1:1000 |
| Anti-STAT3 | 9132 | Cell Signaling Technology | 1:1000 |
| Anti-pTyr690-STAT2 | 88410 | Cell Signaling Technology | 1:1000 |
| Anti-STAT2 | 72604 | Cell Signaling Technology | 1:1000 |
| Anti-pSer176/180-IKKα/β | 9740 | Cell Signaling Technology | 1:1000 |
| Anti-IKKα | 2682 | Cell Signaling Technology | 1:1000 |
| Anti-Tubulin | sc-5286 | Santa Cruz | 1:5000 |

**Table S2： The primer sequences for real-time PCR**

| **Gene name** | **Primer sequence** |
| --- | --- |
| TNF-α Forward | CAGGCGGTGCCTATGTCTC |
| TNF-α Reverse | CGATCACCCCGAAGTTCAGTAG |
| IL-6 Forward | TAGTCCTTCCTACCCCAATTTCC |
| IL-6 Reverse | TTGGTCCTTAGCCACTCCTTC |
| IL-1β Forward | GAAATGCCACCTTTTGACAGTG |
| IL-1β Reverse | TGGATGCTCTCATCAGGACAG |
| CXCL1 Forward | CACAGGGGCGCCTATCGCCAA |
| CXCL1 Reverse | CAAGGCAAGCCTCGCGACCAT |
| CXCL2 Forward | CCAACCACCAGGCTACAGG |
| CXCL2 Reverse | GCGTCACACTCAAGCTCTG |
| CXCL3 Forward | CAGCCACACTCCAGCCTA |
| CXCL3 Reverse | CACAACAGCCCCTGTAGC |
| CXCL10 Forward | CCAAGTGCTGCCGTCATTTTC |
| CXCL10 Reverse | GGCTCGCAGGGATGATTTCAA |
| CCL2 Forward | TTAAAAACCTGGATCGGAACCAA |
| CCL2 Reverse | GCATTAGCTTCAGATTTACGGGT |
| CCL3 Forward | TTCTCTGTACCATGACACTCTGC |
| CCL3 Reverse | CGTGGAATCTTCCGGCTGTAG |
| IL-17A Forward | TTTAACTCCCTTGGCGCAAAA |
| IL-17A Reverse | CTTTCCCTCCGCATTGACAC |
| IFN-γ Forward | ATGAACGCTACACACTGCATC |
| IFN-γ Reverse | CCATCCTTTTGCCAGTTCCTC |
| IFN-β Forward | CAGCTCCAAGAAAGGACGAAC |
| IFN-β Reverse | GGCAGTGTAACTCTTCTGCAT |
| GAPDH Forward | ATGCCTGCTTCACCACCTTCT |
| GAPDH Reverse | CATGGCCTTCCGTGTTCCTA |
