# Supplementary figures and images for: Bioactive compound C498-0670 alleviates LPS-induced sepsis via JAK/STAT and NFκB signaling pathways
Source: Front Immunol. 2023 Apr 14;14:1132265. doi: 10.3389/fimmu.2023.1132265 (PMC10140310; doi:10.3389/fimmu.2023.1132265)

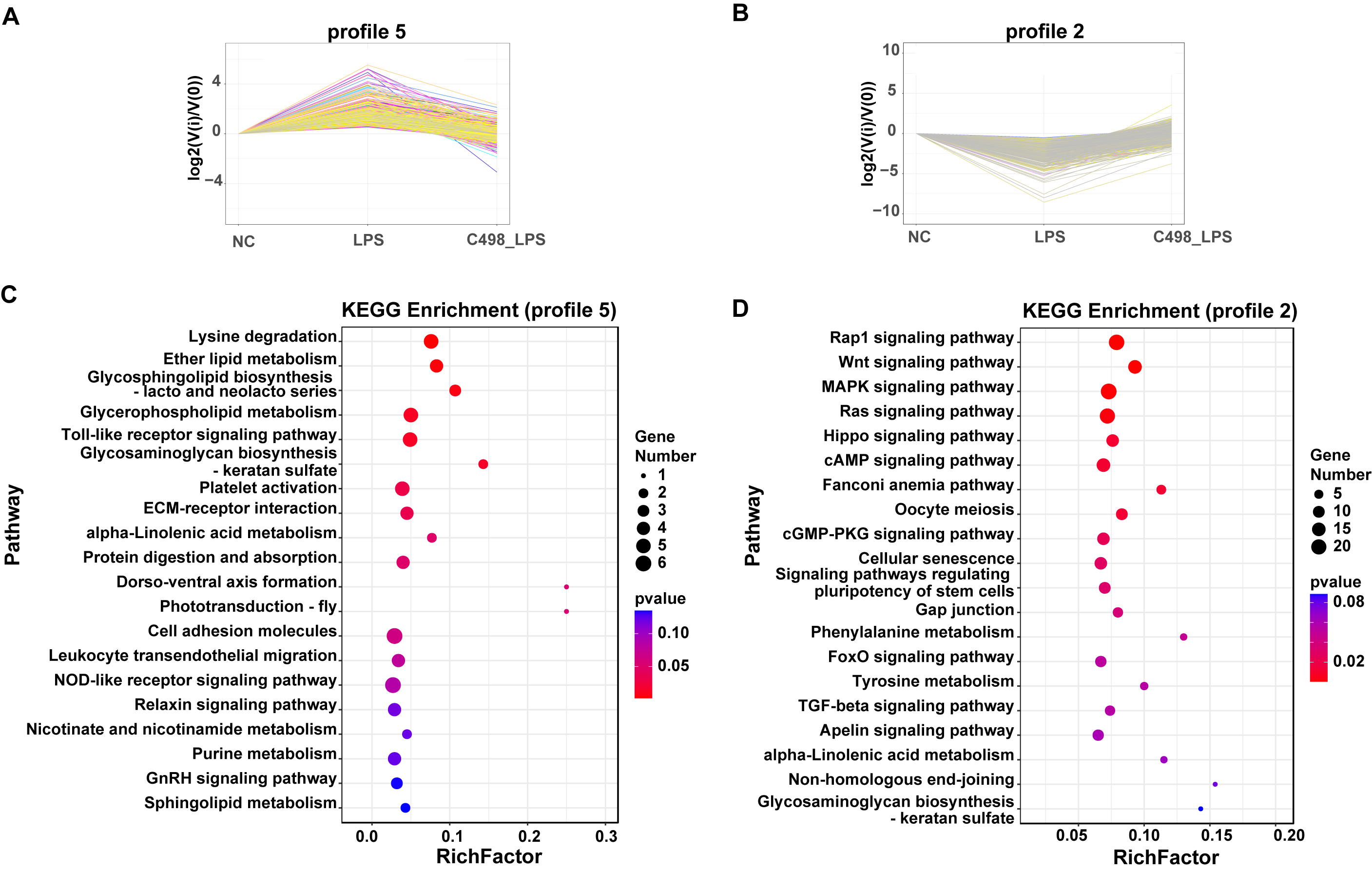

Supplement: Supplementary file 2 [file DataSheet_2.zip › FIGURE S4.tif]

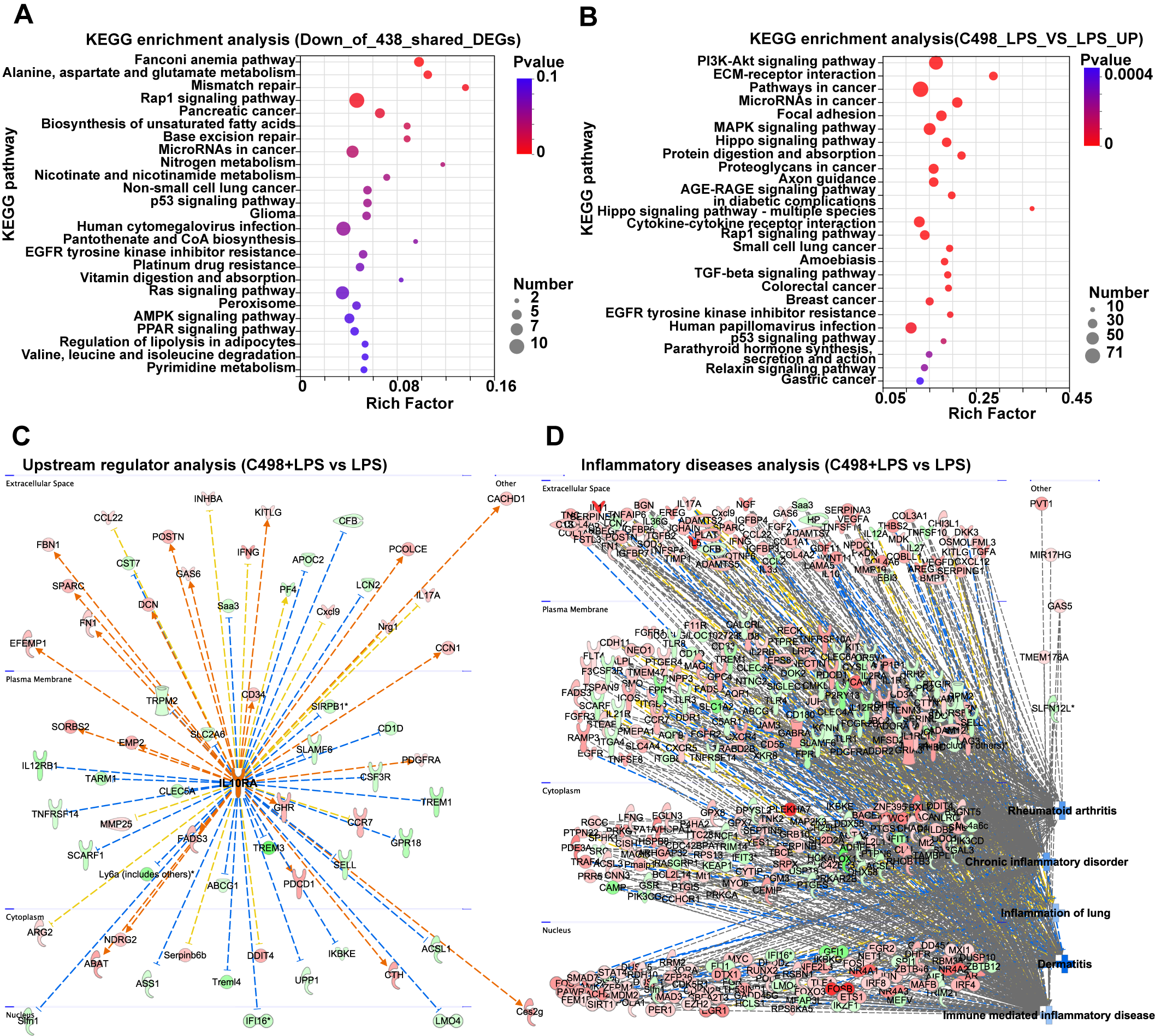

Supplement: Supplementary file 2 [file DataSheet_2.zip › FIGURE S3.tif]

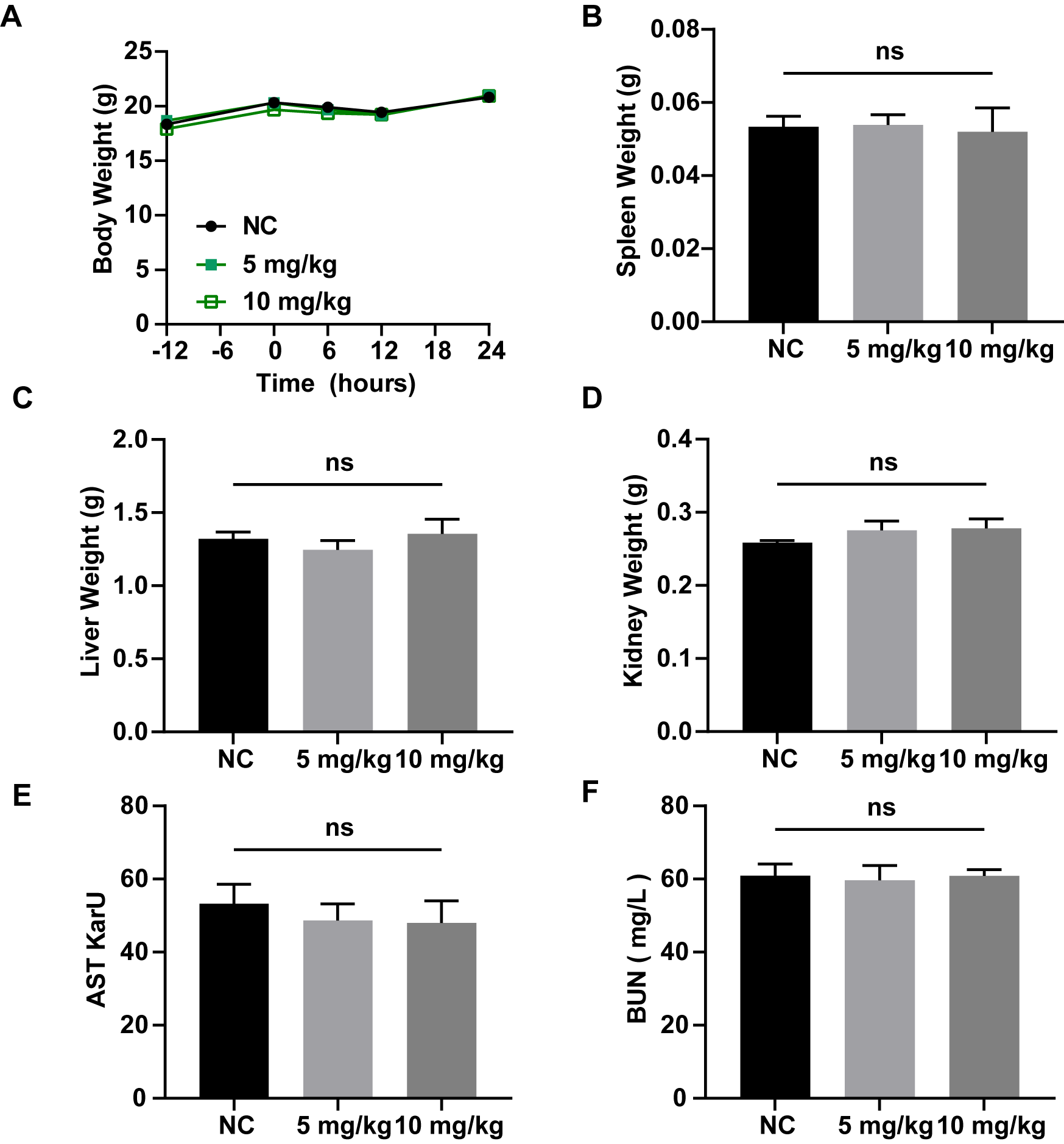

Supplement: Supplementary file 2 [file DataSheet_2.zip › FIGURE S6.tif]

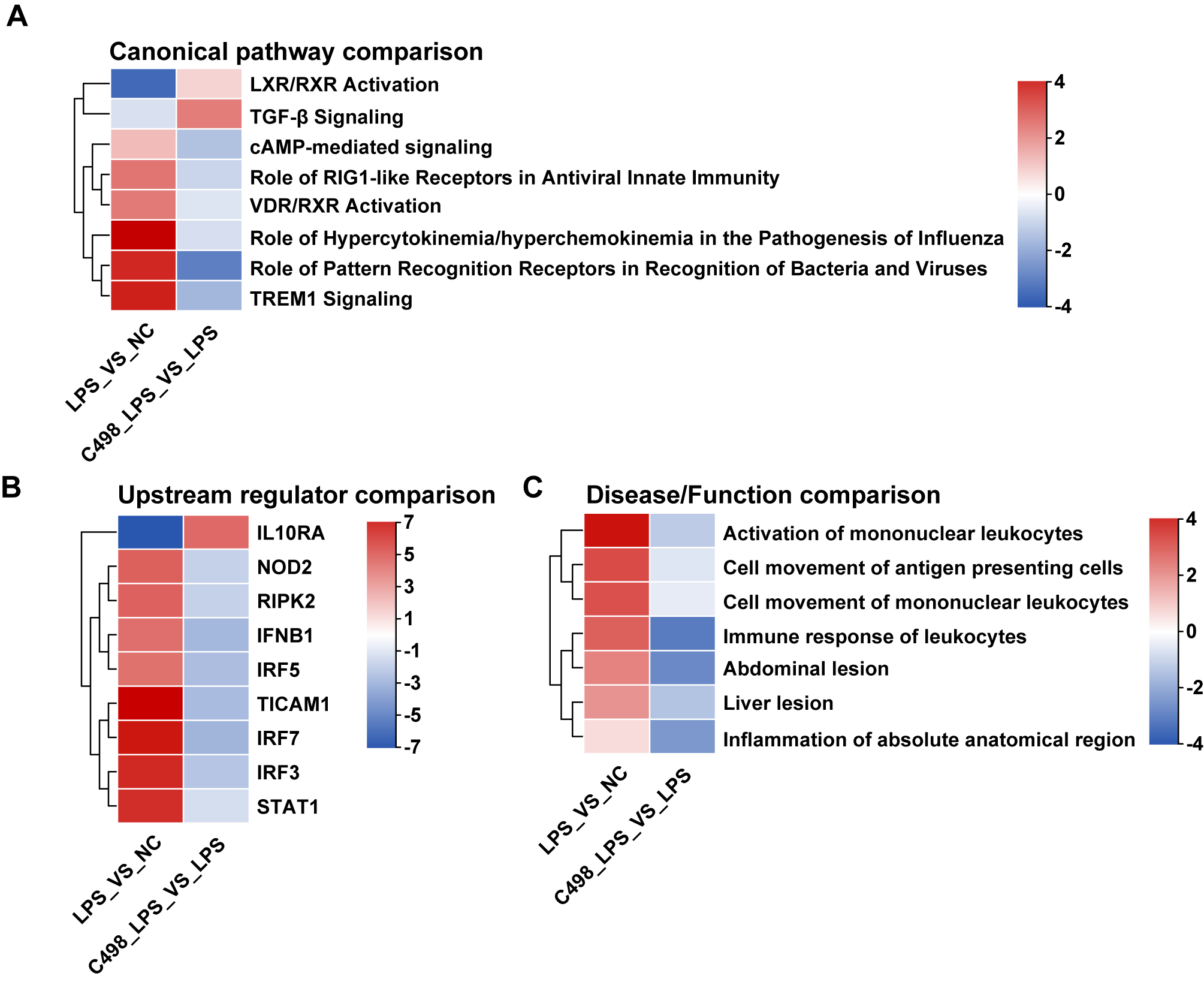

Supplement: Supplementary file 2 [file DataSheet_2.zip › FIGURE S5.tif]

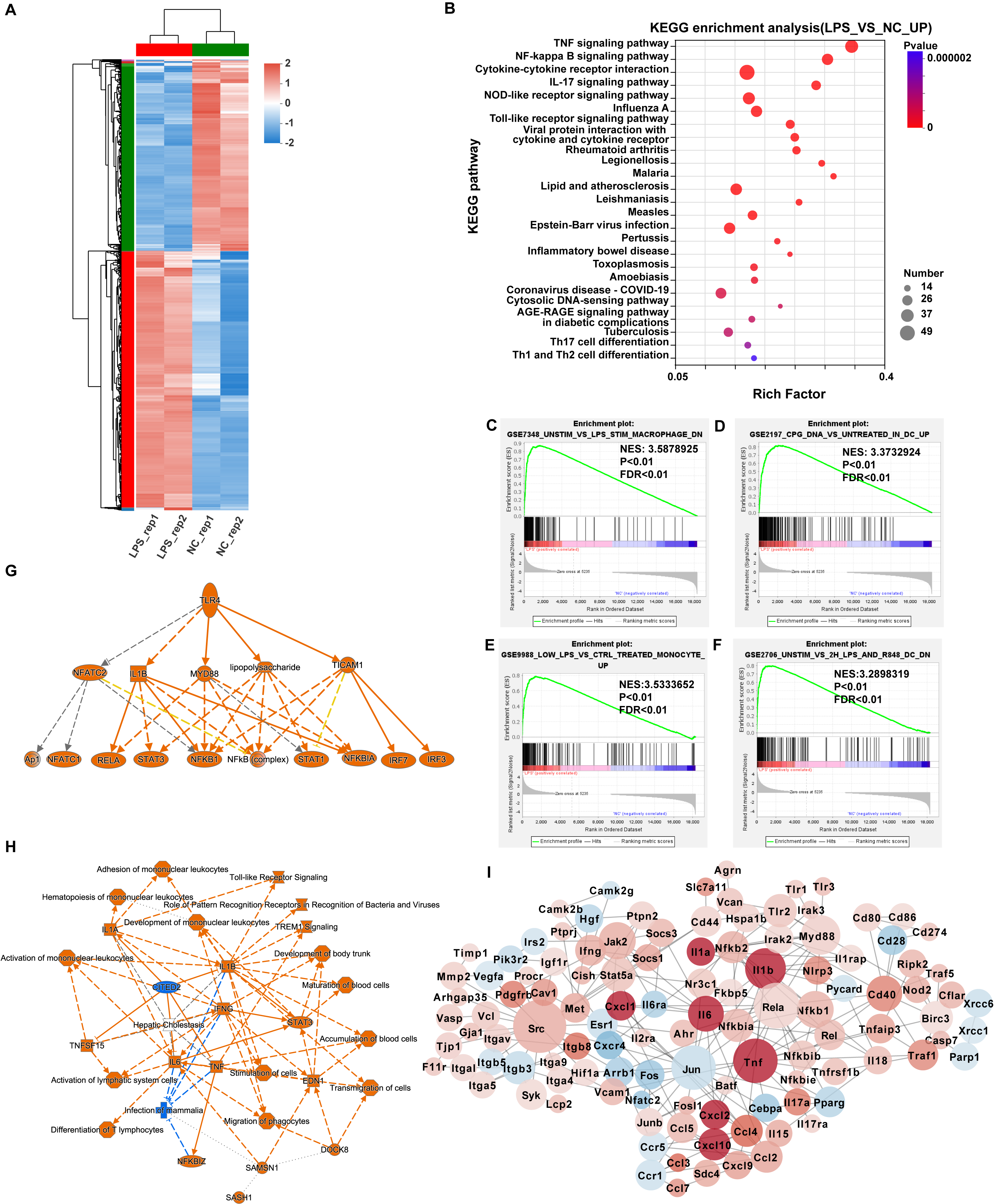

Supplement: Supplementary file 2 [file DataSheet_2.zip › FIGURE S2.tif]

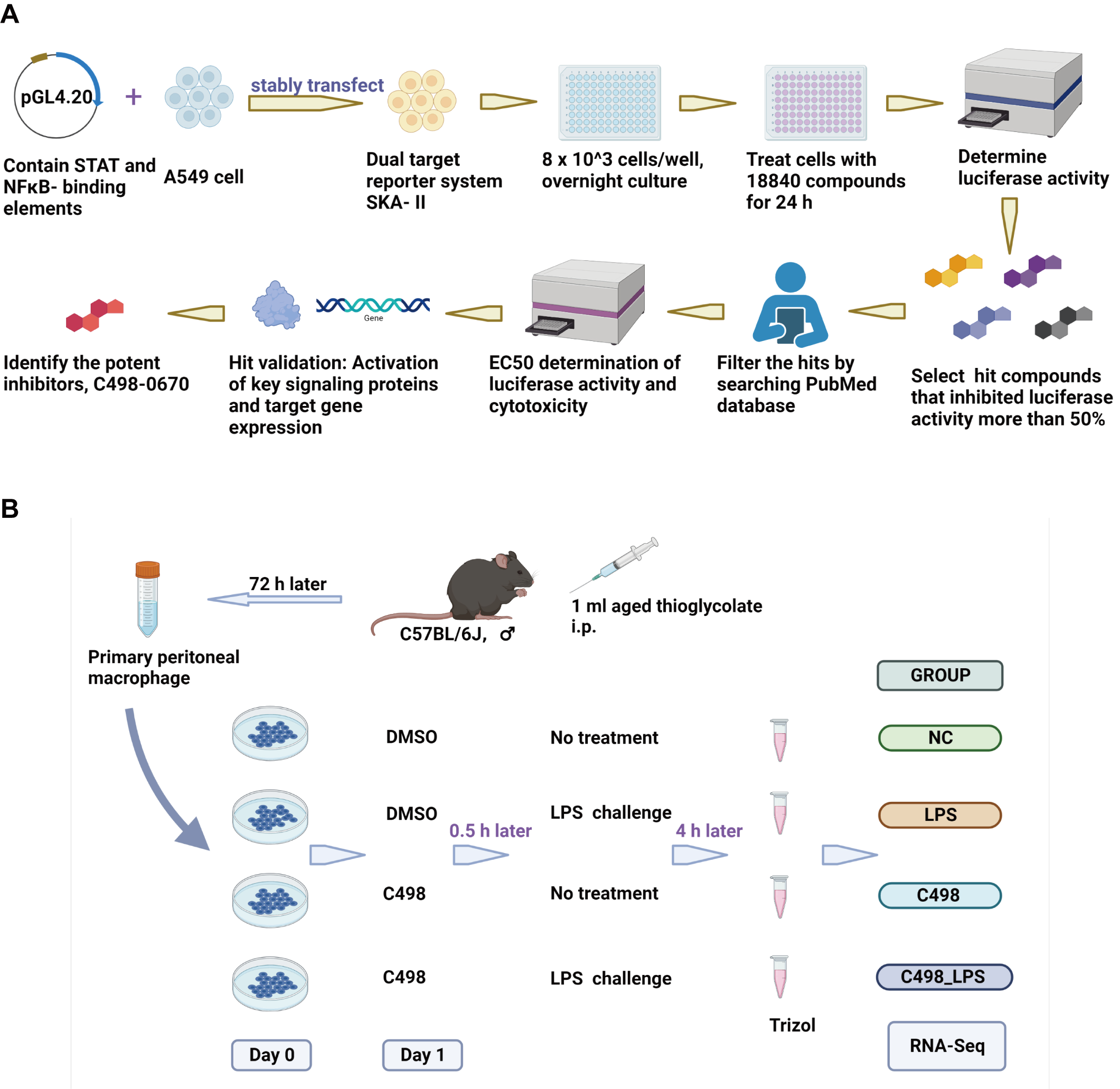

Supplement: Supplementary file 2 [file DataSheet_2.zip › FIGURE S1.tif]
